# Inhibition of αIIbβ3 Ligand Binding by an αIIb Peptide that Clasps the Hybrid Domain to the βI Domain of β3

**DOI:** 10.1371/journal.pone.0134952

**Published:** 2015-09-02

**Authors:** Wen Hwa Lee, Elisabeth Schaffner-Reckinger, Demokritos C. Tsoukatos, Kelly Aylward, Vassilios Moussis, Vassilios Tsikaris, Paraskevi Trypou, Marion Egot, Dominique Baruch, Nelly Kieffer, Christilla Bachelot-Loza

**Affiliations:** 1 SGC, University of Oxford, Oxford, United Kingdom; 2 Laboratoire de Biologie et Physiologie Intégrée, (CNRS/GDRE-ITI), University of Luxembourg, Luxembourg City, Luxembourg; 3 Department of Chemistry, Section of Organic Chemistry and Biochemistry, Ioannina, Greece; 4 Inserm UMR_S 1140, Faculté de pharmacie, Paris, France; 5 Université Paris Descartes, Sorbonne Paris Cité, Paris, France; 6 CNRS-LIA124, Sino-French Research Center for Life Sciences and Genomics, Rui Jin Hospital, Jiao Tong University School of Medicine, Shanghai, China; Royal College of Surgeons, IRELAND

## Abstract

Agonist-stimulated platelet activation triggers conformational changes of integrin αIIbβ3, allowing fibrinogen binding and platelet aggregation. We have previously shown that an octapeptide, ^p1^YMESRADR^8^, corresponding to amino acids 313–320 of the β-ribbon extending from the β-propeller domain of αIIb, acts as a potent inhibitor of platelet aggregation. Here we have performed *in silico* modelling analysis of the interaction of this peptide with αIIbβ3 in its bent and closed (not swing-out) conformation and show that the peptide is able to act as a substitute for the β-ribbon by forming a clasp restraining the β3 hybrid and βI domains in a closed conformation. The involvement of species-specific residues of the β3 hybrid domain (E356 and K384) and the β1 domain (E297) as well as an intrapeptide bond (^p^E315-^p^R317) were confirmed as important for this interaction by mutagenesis studies of αIIbβ3 expressed in CHO cells and native or substituted peptide inhibitory studies on platelet functions. Furthermore, NMR data corroborate the above results. Our findings provide insight into the important functional role of the αIIb β-ribbon in preventing integrin αIIbβ3 head piece opening, and highlight a potential new therapeutic approach to prevent integrin ligand binding.

## Introduction

Integrin αIIbβ3, the platelet fibrinogen and von Willebrand factor (vWF) receptor, plays a key role in hemostasis and thrombosis, by promoting platelet aggregation and thrombus formation at sites of vascular injury. As circulating platelets in blood are constantly exposed to high concentrations of fibrinogen, αIIbβ3 ligand binding has to be tightly controlled to prevent inappropriate thrombus formation. Integrin αIIbβ3 is therefore maintained in a constitutive low-affinity state, and agonist-induced platelet stimulation is required to convert αIIbβ3 from a low- to a high-affinity state, able to bind ligands [1].

Unravelling the molecular mechanisms that regulate platelet integrin αIIbβ3 activation has been the focus of intense research. Initial structural information came from electron microscopy (EM) pictures of purified αIIbβ3 showing its particular shape with a globular head piece connected to two flexible stalks [2]. The 3.1Å crystal structure of the ectodomain of integrin αvβ3 [3], and later of αIIbβ3 [4, 5], revealed that the ligand-binding globular head piece is composed of the N-terminal part of both α and β subunits, comprising the β-propeller of the α subunit associated to the βI and hybrid domains of the β subunit. The flexible stalks or legs comprise multiple domains, corresponding to an Ig-like “thigh” and two β-sandwich “calf” domains for the α subunit, and one PSI, four EGF and one β-tail (βTD) domains for the β subunit. An unexpected initial finding however was the bent, V-shaped conformation of the αvβ3 receptor with a sharp knee-like kink in the middle section of both legs, bringing the headpiece into intimate contact with the lower leg domains.

On the basis of data obtained from crystal structures of αIIbβ3 [4, 5], epitope mapping with anti-αIIb, -β3 or -αIIbβ3 antibodies [6, 7], as well as EM and cryo-electron microscopy (cryo-EM) pictures [8–11], it is now largely accepted that the bent-closed conformation represents the low-affinity, resting state of αIIbβ3. Integrin affinity upregulation relies on several major conformational changes, namely integrin extension at the knees, leg separation, and a β3-subunit swing-out motion at the interface between the βI and hybrid domains, converting the headpiece from a closed to an open conformation, thus facilitating ligand binding by the headpiece [12, 13].

Experiments based on mutant αIIbβ3 receptor function or LIBS antibody bindings have shown that the low-affinity state of β3 integrins is stabilized by several clasps, two of which exist at the transmembrane and cytoplasmic face of the receptor [14–16]. Additional contacts also exist in multiple points along the α and β extracellular legs [6, 15], which might be involved in modulating the transition through different conformations between the inactive and active states of integrins. More importantly however, a clasp in the headpiece, involving the β-ribbon of the αv β-propeller domain (residues 301–308) or the corresponding αIIb domain (segment 313–320) appear to play a major role in preventing the swing-out motion of the β3 hybrid domain [5, 17]. This is consistent with previous suggestions of the existence of such a clasp in integrin αIIbβ3 [4], together with results from targeted molecular dynamic (TMD) studies [18]. These results have been confirmed by Kamata et al. by engineering disulphide bonds between these regions resulting in inhibition of ligand binding [19], and further corroborated using new engineered disulphide bond mutant receptors [20]. Also, peptides corresponding to the αv or the related αIIb sequence as well as the β3 sequence of this clasp promoted integrin activation [17]. Intriguingly, the reported αIIb peptide (^p^MESRADRK) almost completely overlaps with an octapeptide ^p^YMESRADR, previously shown by us to exert an inhibitory effect on αIIbβ3 integrin function by blocking fibrinogen binding to αIIbβ3 [21, 22]. Considering these apparently contradictory data, we have performed *in silico* structural modelling of our octapeptide with αIIbβ3 in its bent-closed conformation (PDB: 3FCS) and used *in silico* as well as experimental data to demonstrate that the αIIb octapeptide, initially thought to interact with fibrinogen [21], substitutes for the αIIb β-ribbon by establishing interactions with both the βI and hybrid domain of β3, thus preventing the swing-out motion of the hybrid domain necessary for head opening and high-affinity ligand binding.

## Materials & Methods

### Peptide Synthesis

Peptides were synthesized on a Wang resin (0.75 meq·g^-1^ resin) using the standard SPPS methodology [23, 24]. Five octapeptides were synthesized: native peptide ^p^YMESRADR, 3 substituted peptides ^p^YMESRAAR, ^p^YMESAADR and ^p^YMESRADA and the “shifted” peptide ^p^MESRADRK.

### Structural Modelling and Analysis

Structural modelling and analysis was performed using the program ICM-Pro v.3.7-2a (www.molsoft.com). All the relevant and available structures were analysed. A combination of superimposition and modelling was performed to generate atomic models of the non-existent integrin conformations, assuming that similar motion ranges are shared by close members of the integrin family. Superimposition was performed (as implemented in ICM) for the domains forming the headpieces followed by tethering of corresponding atoms from homologous and/or identical domains. The tether energies were minimised whilst phi/psi torsion angles of residues found at interdomain hinge regions were made to rotate within allowed regions. Next, side-chain clashes were solved using local energy minimisation cycles. The remaining clashes were solved through Monte-Carlo searches followed by local energy minimisation cycles. The length of the Monte-Carlo searches was defined by the algorithm implemented in ICM.

### Platelet Preparation

Venous blood from informed healthy donors was obtained from the French blood bank institute (EFS) according to the agreement between INSERM and EFS (CPSL C UNT—06/EFS/029). During the medical examination preceding blood donation, the medical doctor of EFS informed the healthy donors that part of their blood could be used for research. All blood samples used in the present study have been provided by healthy donors, which signed the agreement. The samples were anonymized at the EFS prior deliverance to the laboratory. According to the French law (L1211-2), this research is considered as a non-interventional research that does not require prior approval of the ethics committee. Blood was collected in Vacutainer tubes containing ACD-A (citric acid-citrate-glucose) (BD Vacutainer, Becton Dickinson, Le Pont de Claix, France) and was centrifuged for 12 min at 210g in presence of PGE1 2 x 10^−7^ M to obtain platelet-rich plasma (PRP). PRP was diluted with washing buffer (103 mM NaCl, 5 mM KCl, 2 mM CaCl_2_, 1 mM MgCl_2_, 5 mM glucose and 36 mM citric acid; pH 6.5) containing PGE1 2 x 10^−7^ M and 0.06 U apyrase/ml (Sigma Aldrich, St. Louis, MO, USA), then centrifuged for 12 min at 1240g to pellet the platelets. This washing step was repeated and the platelets were finally resuspended at 2.5 x 10^8^ per ml in reaction buffer (10 mM Hepes, 140 mM NaCl, 3 mM KCl, 5 mM NaHCO_3_, 0.5 mM MgCl_2_, 10 mM glucose and 2 mM CaCl_2_; pH 7.4).

### Flow Cytometry

Flow cytometry experiments were performed using a Becton Dickinson FACSort. Platelets were preincubated 5 min with either vehicle (NaCl 0.9%), native or substituted octapeptides (1 mM). When indicated, a further incubation was performed in the presence of RGDS (1 mM). Platelets were activated 10 min with thrombin 2.5 U/ml (Sigma Aldrich, St. Louis, MO, USA). Then, the samples were diluted 1:10 in reaction buffer and incubated with both fluorescein isothiocyanate (FITC)-conjugated PAC1 antibody (PAC1-FITC from Becton Dickinson, San Jose, CA, USA) and phycoerythrin (PE)-conjugated anti-CD62P (from Beckman Coulter, Brea, CA, USA) to measure αIIbβ3 activation and P-selectin expression, respectively. Platelet activation induces the secretion of granule contents, consecutively secreted fibrinogen can bind to activated integrin. Therefore, fibrinogen binding was measured by single-colour flow cytometry using FITC-conjugated rabbit polyclonal anti-human fibrinogen antibody. Exposure of LIBS was evaluated performing mAb AP5 binding (generous gift of Dr T. J. Kunicki, The Scripps Research Institute, La Jolla, CA, USA), and revealed with PE-labelled anti-mAb antibody. In each experiment, controls were run with the corresponding isotype control. Single intact platelet gate was identified on resting sample by their characteristic profile on a right angle scatter (SSC) and forward angle scatter (FSC) plot. A total of 10,000 platelet events were recorded. Results were expressed as the mean fluorescence intensity of resting or thrombin-activated platelets preincubated with or without RGDS and/or octapeptides relative to the basal condition corresponding to resting platelets preincubated with vehicle.

### Aggregation

Washed platelet aggregation was measured with a PAP-8E Biodata optical aggregometer. Platelets were pre-incubated with octapeptide (^p^YMESRADR, ^p^YMESRAAR or ^p^MESRADRK) (75 to 500 μM) or vehicle for 5 min at 37°C with stirring (1200 rpm). Thrombin was then added and aggregation was monitored for 5 min as the change in light transmittance. Results are expressed as the percentage of maximum aggregation ± SEM.

### cDNA Constructs, Transfection and Generation of Stable Cell Clones

The cDNAs encoding αIIbR317A/D319A/R320A (αIIb_3M_) and β3K384A (β3_1M_) were generated using the Quikchange site-directed mutagenesis method (Agilent Technologies, Santa Clara, CA, USA) and the pcDNA3.1(-)Neo-αIIbwt and pcDNA3.1(-)Zeo-β3wt as templates. The nucleotide sequence of the generated mutants was verified by automated sequencing. Chinese hamster ovary (CHO) cell (CRL 9096, American Type Culture Collection, Rockville, MD) transfections were performed using lipofectAMINE (Life Technologies Europe B.V., Ghent, Belgium) as previously described [25], and positive transfectants were screened by flow cytometry for cell surface expression of the recombinant mutant αIIbβ3 complex using an αIIbβ3 complex-specific mAb, Pl2-73 (kind gift from Dr Cécile Kaplan, Institut National de la Transfusion Sanguine, Paris, France), and subcloned by limiting dilution to establish stable cell clones. Finally, we selected the cell clones having a surface expression of the mutant αIIbβ3 receptors comparable to the cell clone expressing the wild type receptor (S1 Fig).

### CHO Cell Adhesion

Cell adhesion was performed using flow conditions and real-time video microscopy as previously described [26]. Briefly, glass coverslips were precoated with 10 μg/ml fibrinogen (from Dako, Glostrup, Denmark), purified and depleted of contaminant fibronectin and vWF, and mounted onto a flow chamber of 200 μm height filled with IMDM. CHO cells resuspended in IMDM at a concentration of 1.25x10^6^/ml were perfused over a fibrinogen-coated coverslip at flow rates from 60–200 μl/min to produce wall shear rates from 30–100 s^-1^. In some experiments, cells were preincubated 20 min with 250–500 μM ^p^YMESRADR or ^p^YMESRAAR peptides before perfusion at 30 s^-1^. Cells were visualized using a 10x Hoffman Modulation Contrast objective together with an inverted microscope. Real-time images were recorded using a charged-coupled-device camera. The number of adherent cells was counted over a 12 min time course using Histolab and Archimed software (Microvision Instruments, Evry, France).

### Statistical Analysis

Results are expressed as mean ± SEM. Differences between samples incubated with vehicle and with peptides were assessed with Wilcoxon test. Cell adhesion mediated by **α**IIb_3M_β3, **α**IIbβ3_1M_ or **α**IIbβ3 WT was compared with Mann-Whitney test (Prism 4.0, GraphPad Software). Degrees of statistical significance are defined as *P<0.05, **P<0.01 and ***P<0.001.

## Results

### Analysis of integrin αIIb β-ribbon residues involved in the clasp preventing αIIbβ3 opening

**Fig 1:**
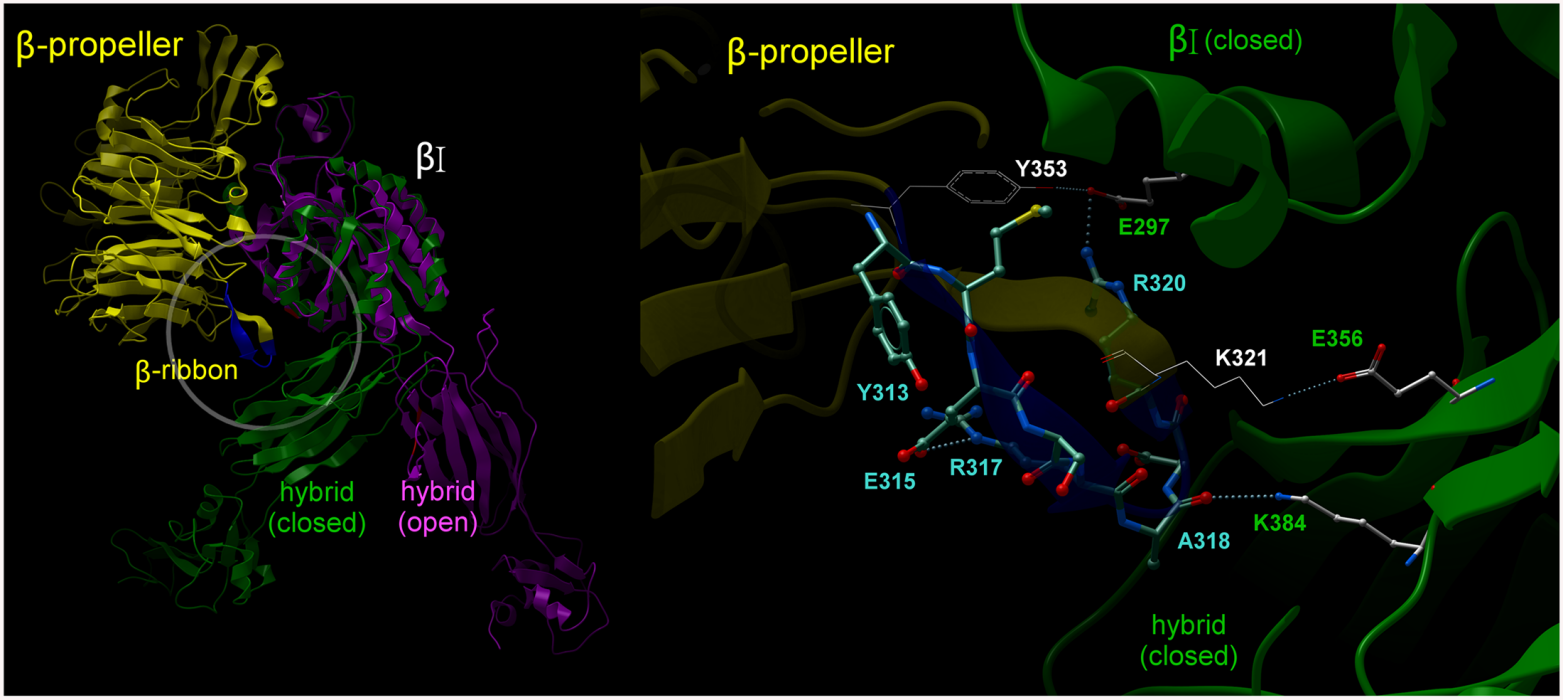
The β-ribbon YMESRADR sequence clasps αIIb to β3 in the bent-closed conformation. [LEFT] Two β3 subunit conformations have been observed in different crystal structures: bent-closed (green) (as seen in the structure of αIIbβ3—3FCS) and extended-open (purple) (as seen in the structure of αIIbβ3—1TY3/2VDK) while the αIIb β-propeller (yellow) position remains unchanged. The latch hairpin (blue, YMESRADR motif) is shown in the structure of the αIIb β-propeller; [RIGHT] Detail of the αIIb latch hairpin interaction with the bent-closed β3 (3FCS). The αIIb β-propeller domain (yellow) containing the β-ribbon and the YMESRADR motif (blue) engages the β3 hybrid domain (green). Two salt-bridges are established across the domains αIIb and β3: R320(αIIb)-E297(βI domain from β3) and K321(αIIb)-E356(hybrid domain from β3). An additional salt-bridge can be observed within the β-ribbon E315(αIIb)-R317(αIIb). In the same vicinity the αIIb β-propeller further engages with the β3 βI-domain through Y353(αIIb)-E297(β3). The interactions indicated above suggest that this region of the αIIb β-propeller plays a role in enforcing the positioning of the βI and hybrid domains when β3 is in its bent-closed conformation. The YMESRADR residues are shown as sticks with the carbon atoms coloured cyan, oxygen in red and nitrogen in blue. Residues from β3 involved in inter-subunit interactions are shown as sticks with white carbon atoms.

The octapeptide ^p^YMESRADR derived from integrin αIIb, previously shown to inhibit platelet aggregation by blocking fibrinogen binding to activated αIIbβ3 [27], corresponds to residues 313–320 of the insert loop (β-ribbon), that extends between the β2 and β3 strands of the W5 blade of the αIIb β-propeller domain [5]. The crystal structure of the bent-closed β3 subunit conformation tightly accommodates this loop in a cleft formed between the βI, hybrid and the third and fourth I-EGF domains of β3. Within the loop, three continuous charged residues establish salt-bridges: E315(αIIb)-R317(αIIb), R320(αIIb)-E297(β3), K321(αIIb)-E356(β3). Also, the carbonyl group of the peptide bond between A318 and D319 (αIIb) from the β-ribbon was seen interacting with K384(β3) (Fig 1).

It is worth mentioning that there are several additional inter-domain interactions between the two integrin subunits outside the region covered by the β-propeller W5 blade loop. One of such interactions, between Y353(αIIb) and E297(β3) positioned in close vicinity of the β-ribbon can be found in αIIbβ3 crystal structures (PDBs 3FCS, 3FCU, 2VDO, 2VDL, 2VDM, 2VDN, 2VDP, 2VDQ, 2VDR, 2VC2, 2VDK).

**Fig 2:**
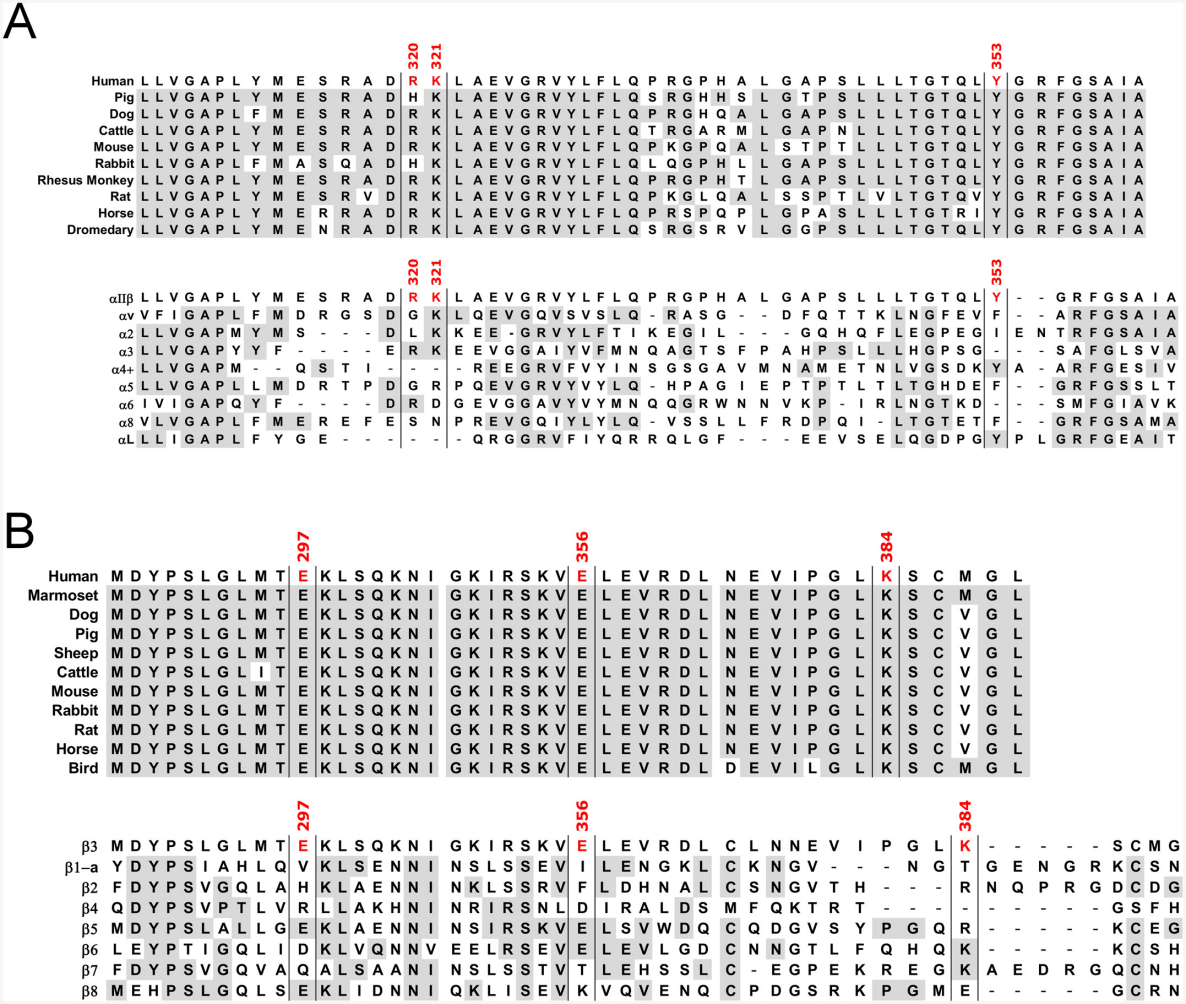
Amino acid sequence alignments. **(A)** Sequence alignments of the region surrounding amino acids 320, 321 and 353 from the human integrin αIIb subunit and αIIb from other species (upper sequences) or with other human α subunits (lower sequences); **(B)** Sequence alignments of the regions surrounding amino acids 297, 356 and 384 from the human integrin β3 subunit and β3 from other species (upper sequences) or with other human β subunits (lower sequences). Conserved residues between human αIIb or β3 and other sequences are shown with light shading.

Alignment of αIIb from several species shows that the β-propeller W5 blade loop is highly conserved, except for R320, which is substituted by histidine in pig and rabbit. It is noteworthy that residues K321 and Y353 are absolutely conserved in all the species included in the analysis (Fig 2A upper alignment) and that the loop is one of the most conserved regions outside the core β-propeller domain, contrasting with the variability seen elsewhere in this domain, and underlining its putative role as a molecular latch.

Similarly, the bent-closed conformation adopted by β3 also revealed a highly conserved surface across 13 species (Fig 2B upper alignment), which coincides with the interfacing region of αIIb and including the β-propeller W5 blade loop. When analysed against homologues, the level of conservation is rather low for the residues implicated in the W5 blade loop (in αIIb) and their counterpart interacting residues found in β3 (Fig 2A–2B lower alignments). This can be interpreted as a result of integrin evolution to diversify the combination between different partnering α and β subunits acquiring novel roles and functions as a consequence.

### Modeling of the interaction of the octapeptide with β3 subunit

**Fig 3:**
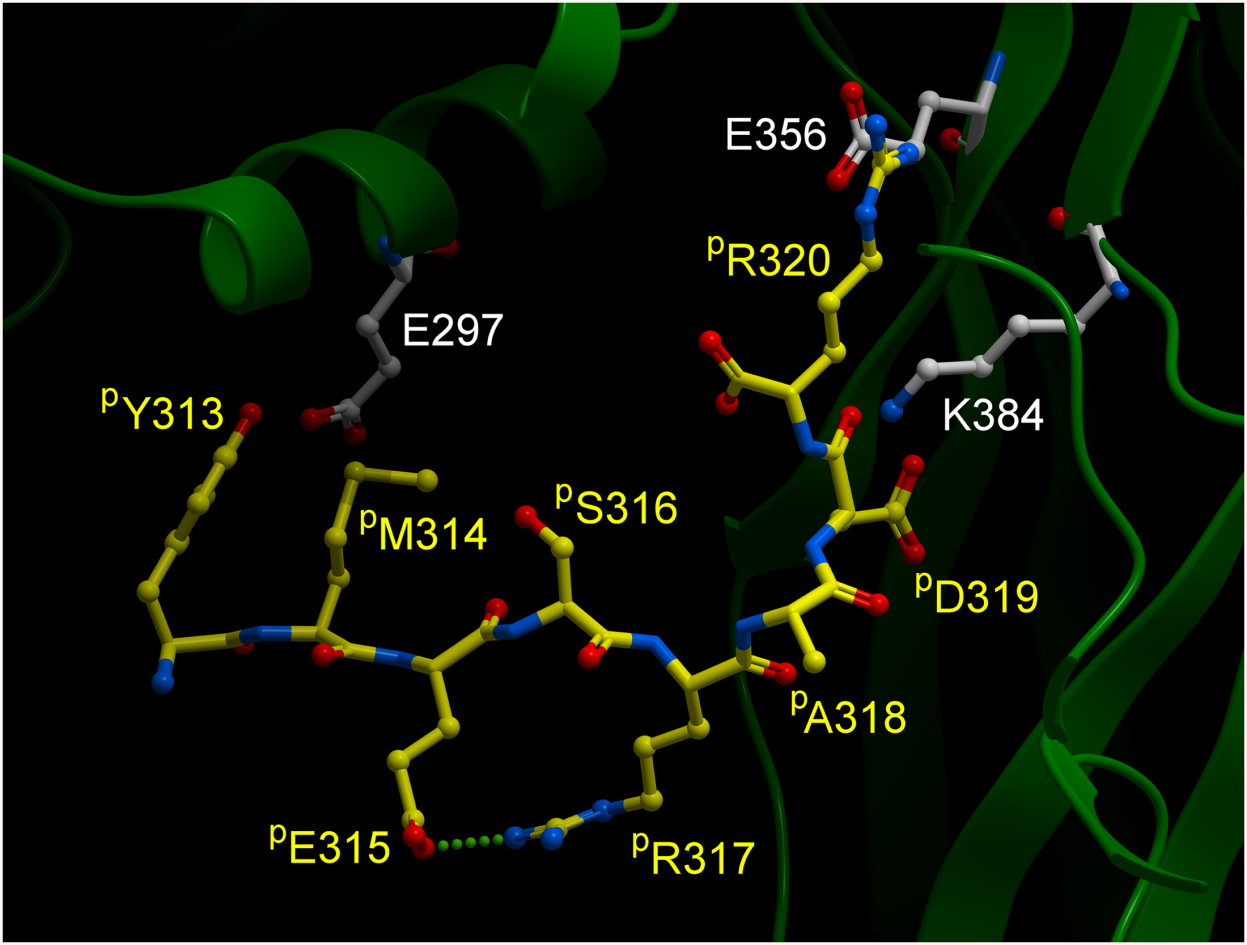
Model of the interaction of the ^p^YMESRADR peptide with the β3 subunit. Conformation adopted by the ^p^YMESRADR peptide to bridge across the βI and hybrid domains of β3. Note that residue ^p^Y313 from the peptide (corresponding to the Y313 from the native hairpin) now replaces Y353 (cf. Fig 1) in interacting with the βI domain. The ^p^E315- ^p^R317 intrapeptide salt bridge is also depicted.

In order to explain the inhibitory effect of the ^p^Y^313^MESRADR^320^ octapeptide on integrin αIIbβ3 activation, we have performed structural modelling to determine how the octapeptide could replace the β-ribbon in restraining the β3 chain in its bent-closed conformation. We have performed *in silico* searches to assess whether a feasible conformation can be achieved, whilst observing the anchoring points used by the octapeptide, derived from the native interactions made by the hairpin. We have inspected the possible solutions and identified the most plausible conformation. In the *in silico* model of the octapeptide, the aforementioned residues involved in salt bridges from β3 are maintained, however these are now formed between the peptide and the hybrid domain (namely ^p^D319(octapeptide)-K384(β3) and ^p^R320(octapeptide)-E356(β3)). Interestingly the intrapeptide salt bridge between ^p^E315-^p^R317 is maintained, despite changes in the backbone geometry. More importantly, this geometry now allows a new interaction to be formed between ^p^Y313 (octapeptide) and E297(β3), completing the restraining of the βI + hybrid domains to the geometry found in the bent-closed conformation (Fig 3).

### Interaction of the octapeptide with the β3 subunit of platelet integrin αIIbβ3

**Fig 4:**
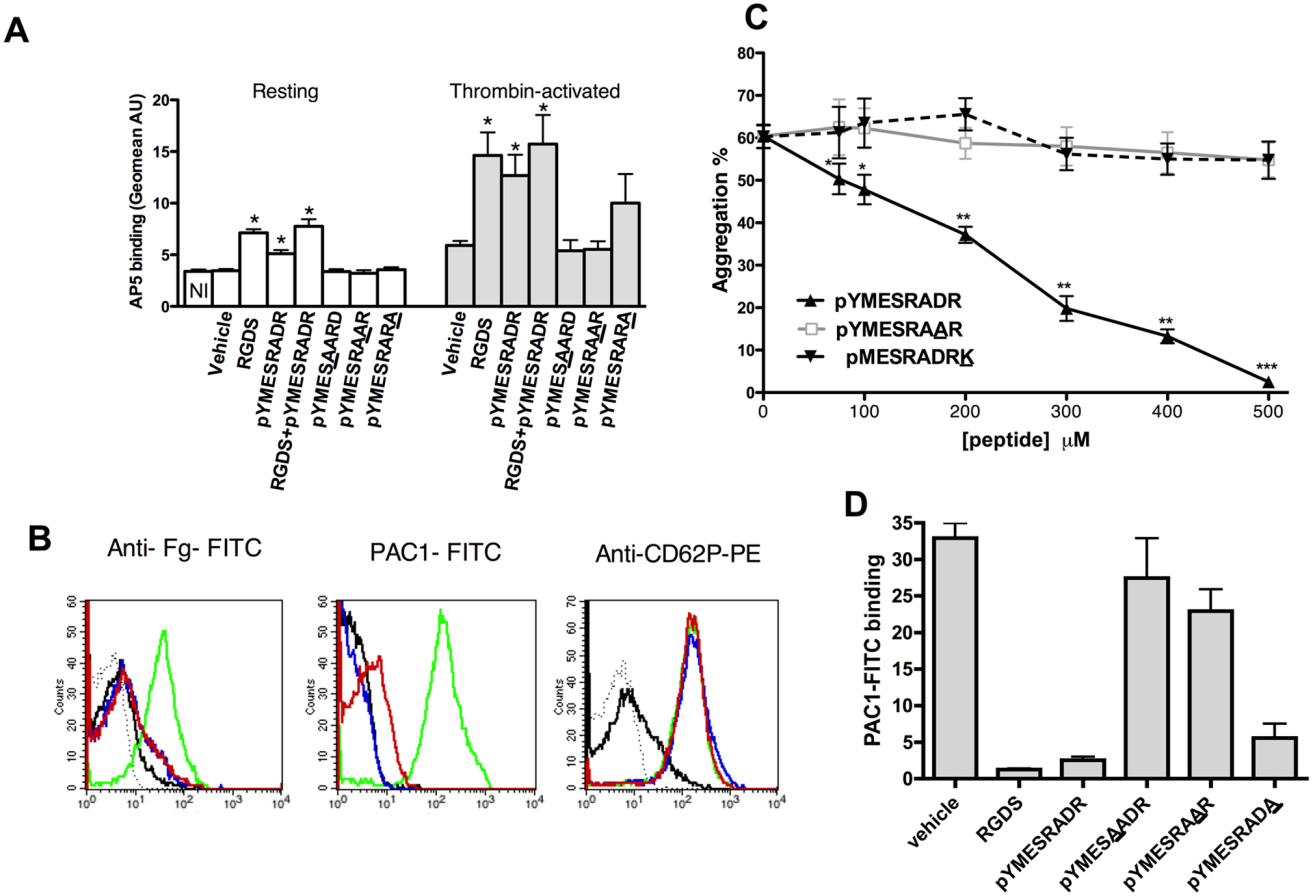
Effect of octapeptides on platelet activation. Washed platelets (2.5x10^8^ platelets/ml) were preincubated with vehicle (NaCl 0.9%) or various peptides (A, B and D: 500 μM) for 5 min. Platelets were then treated with 0.1 U/ml thrombin (activated) or vehicle (resting) as described in “Experimental Procedures”. **(A)** LIBS (AP5 mAb) expression induced by vehicle, RGDS, ^p^YMESRADR, RGDS + ^p^YMESRADR, 317-substituted octapeptide (^p^Y^313^MESAADR^320^), 319-substituted octapeptide (^p^Y^313^MESRAAR^320^) or 320-substituted octapeptide (^p^Y^313^MESRADA^320^) on resting or thrombin-activated platelets. AP5 binding was quantified by determining the fluorescence intensity (Geomean UA) of anti-mAb-phycoerythrin binding (n = 6; mean ± SEM). Isotype control binding (NI, first column) was performed in each experiment; **(B)** Flow cytometry analysis of anti-fibrinogen-FITC, PAC1-FITC, anti-CD62P-PE or isotype control (dashed black lines) antibody binding on resting (black lines) or activated platelets preincubated with vehicle (green lines), ^p^YMESRADR octapeptide (red lines), or RGDS (blue lines); **(C)** Inhibitory effects of octapeptides (^p^Y^313^MESRADR^320^: black; substituted ^p^Y^313^MESRAAR^320^: grey or ^p^M^314^ESRADRK^321^: dashed) on human washed platelet aggregation induced by thrombin (0.1 U/ml). Platelets were preincubated with vehicle (peptide 0 μM) or various concentrations of octapeptides and stimulated with thrombin for 5 min. Aggregation is expressed as a percentage of maximal light transmission measured at 5 min. Each point represents the mean (± SEM) of at least 4 experiments, *P<0.05, **P<0.01, ***P< 0,001 versus untreated platelets (peptide 0 μM); **(D)** Mean fluorescence intensity of PAC1-FITC binding on thrombin-activated platelets preincubated with vehicle, RGDS, ^p^YMESRADR or with 317 substituted octapeptide (^p^YMESAADR), 319 substituted octapeptide (^p^YMESRAAR) or 320 substituted octapeptide (^p^YMESRADA).

To further test the hypothesis that the octapeptide inhibits platelet activation by direct interaction with the β3 subunit, we investigated its ability to induce the exposure of LIBS (ligand-induced binding site) neo-epitopes on β3. LIBS epitopes were initially identified on αIIbβ3 following binding of peptide antagonists, such as RGDS, or certain antibodies [28] and are considered as markers of multiple conformational changes of αIIbβ3 that occur during the transition of αIIbβ3 from its unliganded-closed to liganded-open conformation [7]. Binding of the β3-specific AP5 LIBS antibody [29] was monitored by flow cytometry. The native octapeptide (Fig 4A) promoted AP5 antibody binding on resting platelets although to a lesser extent than RGDS did, whereas it was as powerful as RGDS to induce AP5 binding on thrombin-activated platelets. Co-treatment with ^p^Y^313^MESRADR^320^ and RGDS did not result in a synergistic effect to induce further AP5 binding. Similar results were also obtained with another β3-specific LIBS antibody, LIBS1, (S2 Fig). Moreover, similarly to RGDS, the octapeptide inhibited fibrinogen binding (Fig 4B left), as well as the binding of PAC1 (Fig 4B middle and Fig 4D), an antibody that specifically recognizes activated αIIbβ3. This binding did not significantly affect platelet secretion, as shown by the absence of any effect on CD62P expression on thrombin-activated platelets (Fig 4B right). These results suggest that the octapeptide does not interact with fibrinogen but inhibits the activation of integrin αIIbβ3. The inhibitory effect of the peptide was also tested in light-transmission aggregometry under stirring conditions and the results show that ^p^Y^313^MESRADR^320^ inhibited platelet aggregation in a concentration-dependent manner (Fig 4C black line).

In order to further validate our model, experiments were designed using variants of the octapeptide in which putative residues interacting with β3 were substituted. The importance of residues ^p^R317, corresponding to the αIIb amino acid involved in intrapeptide salt bridge R317-E315 (Fig 3), and ^p^D319, was demonstrated by the loss of activity of ^p^R317A or ^p^D319A substituted octapeptides when tested by measuring AP5 binding (Fig 4A), platelet aggregation (Fig 4C grey line) and PAC1 binding (Fig 4D). In contrast, ^p^R320A decreased only partially the ability of the peptide to induce AP5 binding (Fig 4A), and consequently, decreased only faintly the peptide inhibitory activity on PAC1 binding (Fig 4D). Similar results were obtained with thrombin-induced fibrinogen binding to platelets (S3 Fig). Finally, we performed aggregation with an octapeptide shifted by one amino acid (^p^M^314^ESRADRK^321^), which, even at 500 μM, did not inhibit platelet aggregation (Fig 4C dashed line). This result shows that amino acid ^p^Y313 is essential for the inhibitory activity of the octapeptide.

The importance of ^p^Y313 for the inhibitory activity of the octapeptide is in line with our modelling results, where ^p^Y313 plays a crucial role in bridging the β-propeller domain across to the βI + hybrid domains. However this requires the octapeptide to adopt a stable conformation in solution similar to the one found in our *in silico* modelling.

**Fig 5:**
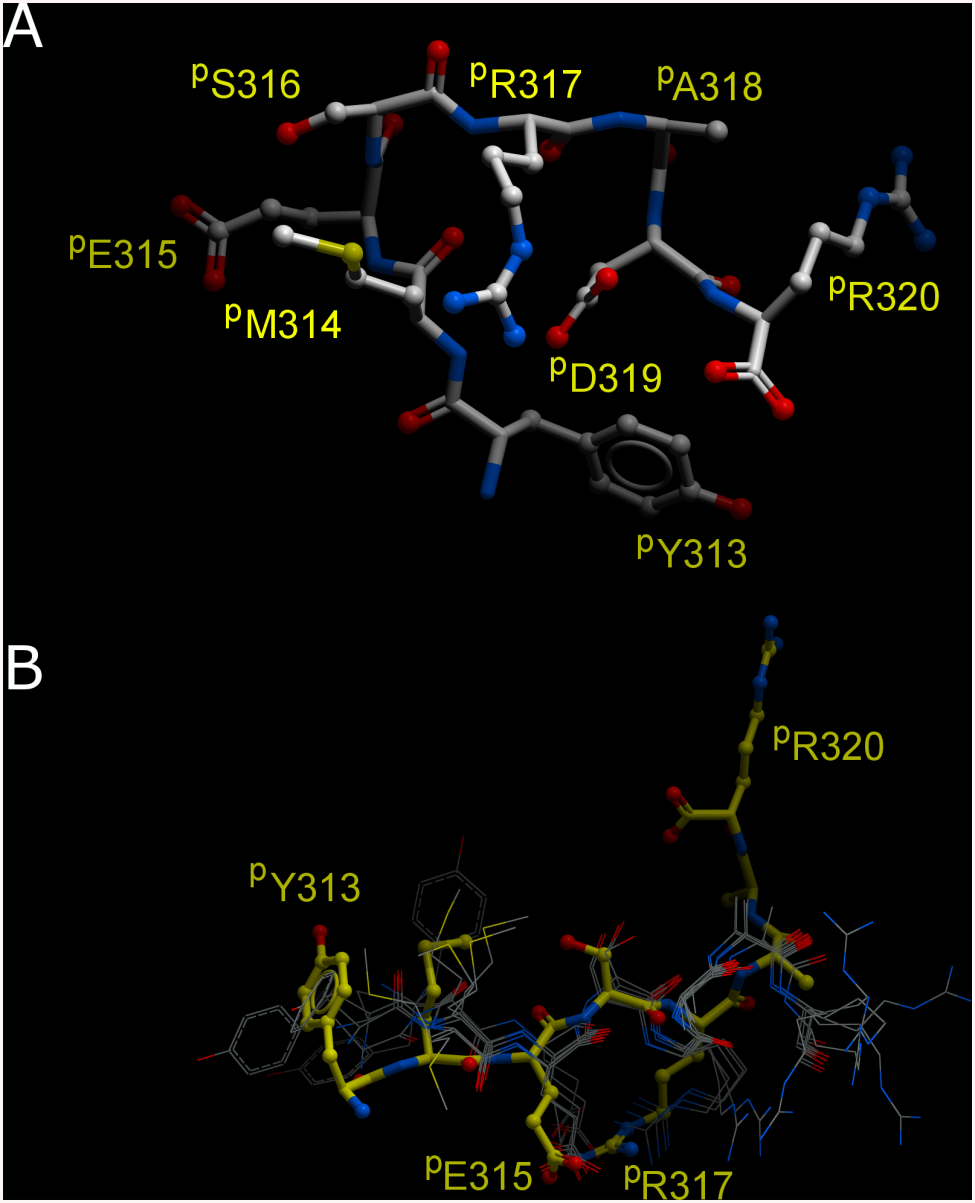
Free solution structures of octapeptides compared to the *in silico* model of ^p^YMESRADR in its inhibitory conformation. **(A)** Solution structure of native octapeptide ^p^YMESRADR determined by NMR; **(B)** *In silico* model of the octapeptide in its inhibitory conformation shown as sticks (as seen in Fig 3) compared to the NMR ensemble of models of the ^p^D319A substituted octapeptide shown as grey wires. Note that the intra-peptide salt-bridge between residues ^p^E315- ^p^R317 is present in both structures.

We then revisited our data from a previous NMR structural and functional characterisation of the native octapeptide and its alanine scanning substitutions [27]. Upon examination of the solution conformational ensembles, it was clear that the backbone conformation of the native peptide in solution was being stabilized via an intra-molecular salt-bridge formed by ^p^R317 and ^p^D319 (Fig 5A). However, more interesting are the NMR data of the peptide with the Alanine substitution at position ^p^D319. Indeed, this substitution induced a new backbone conformation with a change of the intra-molecular salt-bridge from ^p^R317- ^p^D319 to ^p^R317- ^p^E315 (Fig 5B), almost identical to the conformation of the native octapeptide in our *in silico* model (Fig 3). This might be rationalised through the fact that most frequently a peptide will rearrange its solution conformation to a different allosteric conformation upon binding/interacting with another macromolecule. The NMR data thus suggest that when residue ^p^D319 is engaged, possibly towards forming the first interaction between the octapeptide and K384 from the β3 subunit, it frees up the intramolecular interaction with R317, which then can engage E315 to compensate for the change (Fig 3) resulting in a more stable conformation of the peptide, in line with our *in silico* model (Fig 5B and Fig 3). This is furthermore reinforced by the fact that the inhibitory activity of the substituted ^p^D319A octapeptide was only 9%.

Once in such stable conformation, ^p^Y313 ends up unexpectedly well-placed to form an interaction with the β3 headpiece E297 residue, effectively restricting to a certain degree both the swing-out and extension movements (Fig 3).

### Mutations of either αIIb or β3 amino acids involved in the clasp result in an increased activity state of recombinant integrin αIIbβ3

We generated cell clones expressing αIIbβ3 with mutations in either αIIb or β3. In the cell clone αIIb_3M_β3, the αIIb subunit had a triple mutation R317A+D319A+R320A, which corresponds to the ^p^RADR sequence considered to be the most crucial for the expression of the inhibitory activity [22]. The mutant αIIb was co-expressed with wild type β3 in CHO cells, while in the cell clone αIIbβ3_1M,_ the β3 subunit had a single K384A mutation and was co-expressed with wild type αIIb. Residue K384 in the αΙΙbβ3 crystal structure is indeed at a favorable distance to form salt bridges with αIIb (Fig 1).

**Fig 6:**
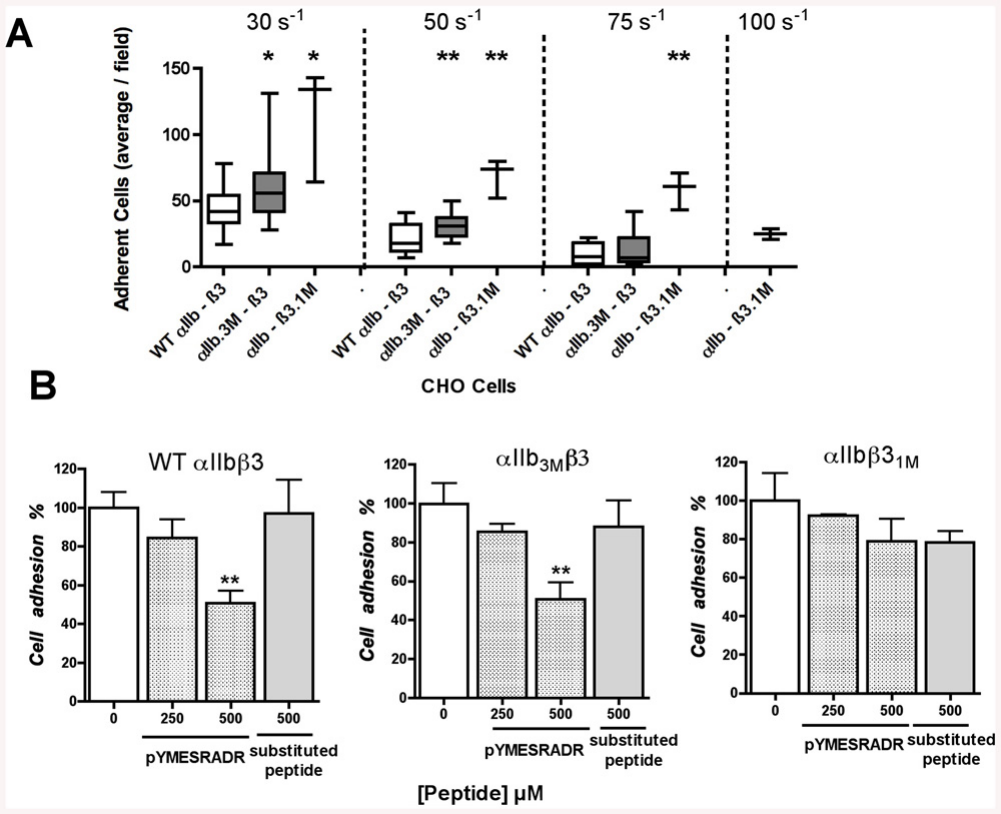
CHO cell adhesion to fibrinogen in flow conditions. CHO cells expressing αIIbβ3 wild type (WT αIIbβ3), mutated on αIIb (αIIb_3M_β3) or on β3 (αIIbβ3_1M_) were perfused over fibrinogen **(A)** at increasing shear rates (30 s^-1^, 50 s^-1^, 75 s^-1^ or 100 s^-1^) or **(B)** were preincubated 20 min with vehicle (0), 250 μM or 500 μM ^p^YMESRADR peptide, or substituted control peptide (^p^YMESRAAR) and then perfused at a shear rate of 30 s^-1^. Adherent cells were counted after 12 min exposure to shear. **(A)** Results are expressed as the number of adherent cells/field, obtained by counting 10 fields. Bars represent mean values and the interquartile range of at least 3 experiments. **(B)** Adhesion of WT (left panel), αIIb_3M_β3 (middle panel) and αIIbβ3_1M_ (right panel) cells in the presence of peptides or vehicle was quantified (as described in methods section). The results are expressed as percentage of adhesion of each clone in presence of peptides relatively to corresponding cells in presence of vehicle (0) normalized to 100%. (n = 6; mean ± SEM). *P<0.05, **P<0.01

It has been established that shear forces can increase the sensitivity of integrin αIIbβ3 adhesive interactions as demonstrated by the shear-dependent increase of adhesion of CHO cells expressing the Pl^A2^ polymorphism of αIIbβ3 [30] and that αIIbβ3 activation is associated with a stabilization of integrin binding to fibrinogen under shear conditions between 30 s^-1^ and 100 s^-1^ [26]. In the present setting, we have demonstrated, as in previous work [26], that increasing shear rates results in decreased cell adhesion mediated by αIIbβ3. Interestingly, cells expressing the mutant integrins exhibited increased adhesion as compared to cells expressing wild type αIIbβ3. Notably, at 100 s^-1^, only cell clone αIIbβ3_1M_ showed residual adhesion. As the receptors displayed similar density and accessibility on the cell surface (S1 Fig), the differences in cell adhesion under shear relate most likely to a different activation state of the αIIbβ3 receptor expressed on the cell surface (Fig 6).

In addition, CHO cell adhesion at the lowest shear rate was determined in the presence of the octapeptide. The ^p^Y^313^MESRADR^320^ native octapeptide strongly impaired adhesion of CHO cells expressing wild type **α**IIbβ3 or mutated αIIb_3M_β3 with a statistically significant effect observed at 500 μM, while the ^p^Y^313^MESRAAR^320^ substituted octapeptide had no effect (Fig 6B). In contrast, native ^p^Y^313^MESRADR^320^ was not able to inhibit αIIbβ3_1M_ CHO cell adhesion onto fibrinogen under shear stress. These results demonstrate that (i) the mutation of αIIb does not impact the binding of the peptide to the integrin in contrast to the β3K384A mutation (αIIbβ3_1M_) and (ii) the residue ^p^D319 in the octapeptide plays a crucial role in the binding of the octapeptide to integrin αIIbβ3.

## Discussion

The aim of our study was to rationalise a model that explains the inhibitory effect on integrin αIIbβ3 activation by the ^p^Y^313^MESRADR^320^, an octapeptide which corresponds to αIIb β-ribbon sequence [4, 5, 18, 22]. Analysis of our *in silico* data have led us to propose a model in which the octapeptide substitutes the αIIb β-ribbon in restraining β3 in its closed conformation, by bridging the βI domain and the hybrid domain of the β3 subunit. This model was then used to guide the design of our experimental methodology.

The proposed model is supported by following experimental data: (i) the octapeptide inhibits platelet aggregation, fibrinogen and PAC1 binding to activated platelets; (ii) alanine replacement or deletion of the amino acids of the octapeptide underline the importance of ^p^D319, ^p^R317, ^p^R320, as well as ^p^Y313 for the inhibitory activity of the peptide. The unexpected involvement of ^p^Y313 may explain the apparent contradictory results with the shifted peptide ^p^M^314^ESRADRK^321^ which was suggested to activate platelets [17]; and finally, (iii) the octapeptide strongly impairs adhesion onto fibrinogen under flow of CHO cells expressing either the wild type receptor or the receptor with mutations in the αIIb β-ribbon. Additionally, the octapeptide is unable to inhibit adhesion of CHO cells expressing the β3K384A mutant, demonstrating the role played by the salt bridge between ^p^D319-K384(β3) in the octapeptide's inhibitory activity.

It is also interesting to note that the heptapeptide LSARLAF, which was designed to bind to residues 315–321 of αIIb activates αIIbβ3 by directly inducing a conformational change in the receptor [31]. Again, this result agrees with our model, with the LSARLAF playing the opposite role of the ^p^YMESRADR peptide. Indeed, by binding to the region in αIIb, which encompasses the β-ribbon, LSARLAF disrupts the interaction between the β-ribbon of αIIb and the hybrid domain of the β3 subunit, thus facilitating its extension. It is likely that full integrin activation by LSARLAF also requires an additional inside-out signalling pathway, as suggested by the study of Pearce *et al* [32].

**Fig 7:**
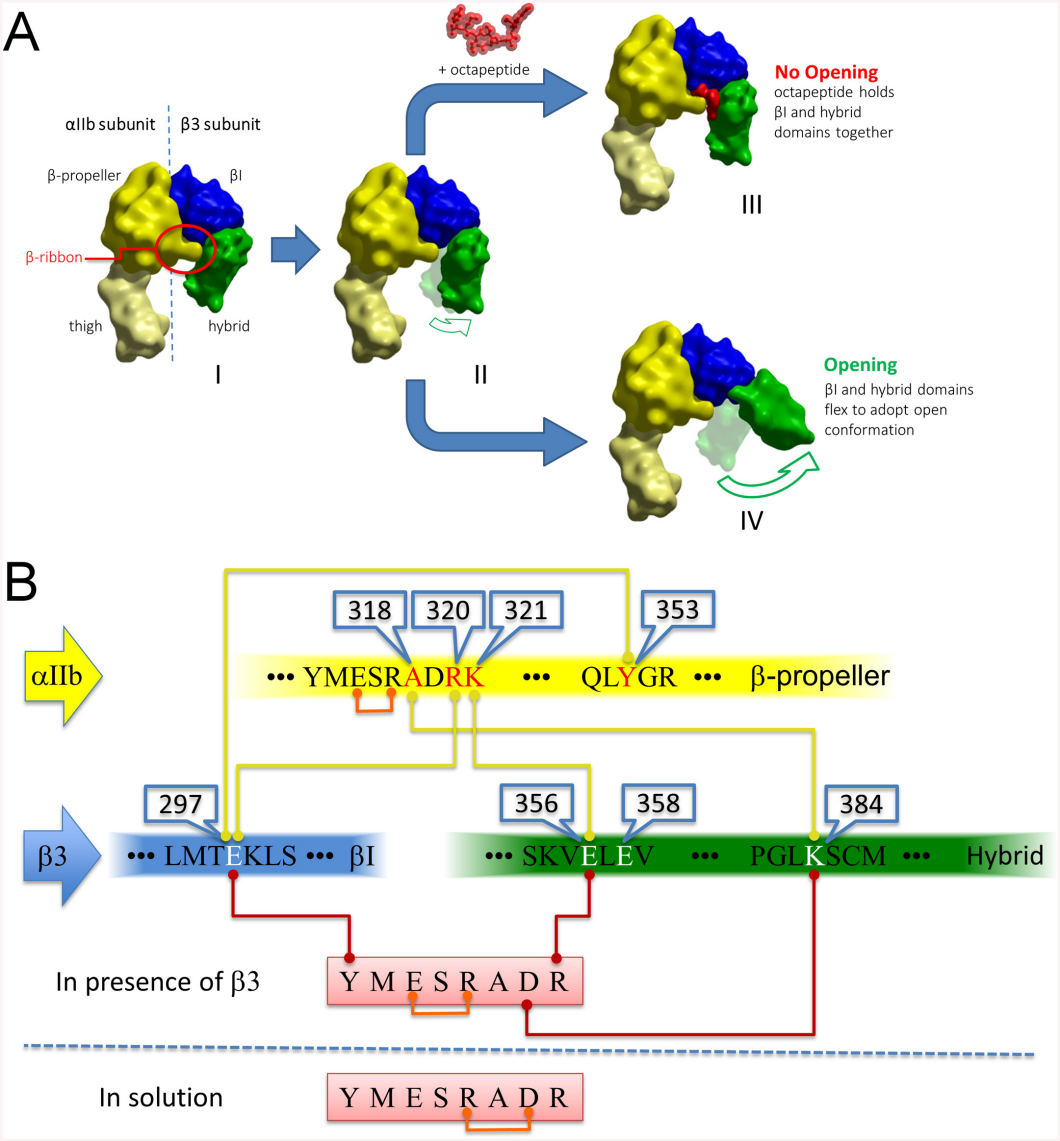
**(A) Integrin extension model and structural rationalisation of inhibition of αIIbβ3 activation by octapeptide** ^**p**^ **YMESRADR.** For clarity only selected domains of the αIIb and β3 subunits are shown. **(I)** αIIbβ3 in the initial bent-closed conformation, the β-ribbon is highlighted in the red circle. (**II)** initial release of the β-ribbon, to be followed by opening of integrin αIIbβ3 **(IV)**. However, if the octapeptide is present **(III)**, it will then substitute the β-ribbon in restraining βI (blue) and hybrid (green) domains, impeding opening and therefore preventing full activation of the integrin; (**B) Schematic representation of αIIbβ3 clasp interactions and the binding positions of the** ^**p**^ **YMESRADR octapeptide.** In the physiological clasp the β-ribbon and residue Y353 in the β-propeller domain (yellow) are linked to the β3 hybrid domain (green) and the βI domain (blue) concomitantly. When the physiological clasp is released, the octapeptide (red) can then engage with the available anchoring points to reestablish the restraint between the βI (blue) and the hybrid (green) domains. Note that the octapeptide (red) is stabilized through different intra-peptide salt-bridges (orange lines): ^p^R317- ^p^D319 when in solution, and ^p^R317- ^p^E315 when bridging across the βI (blue) and the hybrid (green) domain. The numbering of the residues is shown in the blue callout boxes.

Taken together, our results indicate that the ^p^Y^313^MESRADR^320^ octapeptide exerts its inhibitory effect on fibrinogen binding through (i) the establishment of ionic bonds with the β3 subunit, involving residues K384 and E356 from the β3 hybrid domain, and (ii) interaction with E297 from the β3 I-like domain, thus bridging and stabilizing the αIIbβ3 headpiece in the closed conformation (Fig 7). This is consistent with the multi-step activation model as proposed by Zhu *et al*. which depicts the process through eight distinct stages [33]. Transition from bent-closed to extended-closed steps occurs without major changes in the integrin αIIbβ3 headpiece. This is followed by opening (swing-out) movement from extended-closed step to extended-open step, which eventually leads to activation. In the swinging-out stage however, during the disengagement of the β-ribbon all the main interaction residues from β3 –namely E297, E356 and K384 become available for binding by the octapeptide, leading to locking of the hybrid and βI domains into an intermediate step resembling the extended-closed conformation (Fig 7)–thus precluding full activation of integrin αIIbβ3.

In fact, this intermediate conformation captured by the binding of the octapeptide might explain our AP5 antibody binding results. Indeed, AP5 has been reported to only bind to αIIbβ3 in its activated state. In our scenario, αIIbβ3 already exposes epitopes recognised by AP5, albeit it is still in an inactive conformation. This is further reinforced by a similar observation made by Cheng *et al*. where a disulphide bond, which was engineered to perform a similar structural role as the one we believe the octapeptide is playing, does not completely inhibit the binding of AP5 induced by Kistrin (a small RGD-containing protein) [20].

However, and given that the octapeptide contains a RAD sequence, one might assume that this sequence could compete with the binding site of the integrin for the RGD domain of fibrinogen. On the other hand, our previous evidence that the octapeptide inhibited rabbit platelet aggregation *ex vivo* and *in vivo*, in fact strongly supports a non-RGD like mechanism of inhibition [34]. Indeed, rabbit platelets have previously been used as a suitable thrombosis model for testing the efficacy of peptides unrelated to RGD, since RGD is inactive on rabbit platelet aggregation and fails to displace fibrinogen from their surface [35, 36]. Moreover, it has been previously demonstrated that RAD peptides do not exhibit any activity and are used as negative controls [37–39]. These are strong arguments, although not definitive, that the octapeptide is probably not interacting with the RGD binding site of the integrin.

In conclusion, our data and those of the literature highlight the important role of the αIIb β-ribbon in maintaining the closed inactive conformation of αIIbβ3 by securing the hinge angle between the βI and the hybrid domain. This precludes conformational changes necessary for the extension of integrin αIIbβ3 to its high-affinity ligand-binding state. The results of the present study lend further support to the above assumption by presenting detailed interactions between residues, and especially by emphasizing the role of Y353(αIIb) in bridging the hybrid domain of β3 with the βI domains of β3. Finally, an allosteric inhibition of αIIbβ3, induced by the octapeptide, may provide an alternative pharmacological approach to the αIIbβ3 RGD-like antagonists. Such allosteric antagonists could reduce the risk of paradoxical receptor activation observed with the cooperative fibrinogen inhibitors [40, 41].

## Supporting Information

S1 Fig Flow cytometry analysis of αIIbβ3 expression in transfected CHO cells. CHO cells expressing the αIIbβ3 wild-type receptor (blue line), the αIIb_3M_β3 mutant (αIIbR317A/D319A/R320A-β3) (green line) or the αIIbβ3_1M_ (αIIb-β3K384A) (red line) were labeled with saturating amounts of anti-αIIb-PE antibody. The isotype control is shown as the thin black line.
(PDF)

S2 Fig Binding of LIBS1 antibody to platelets. LIBS1 or control antibody (NI) binding on resting or thrombin-activated platelets pre-incubated with vehicle, RGDS or ^p^YMESRADR peptides. Bound LIBS1 was revealed with PE- anti-mAb antibody and quantified on Becton Dickinson FACSort cytometer.
(PDF)

S3 Fig Effect of octapeptides on fibrinogen-binding to platelets. Washed platelets (2.5x10^8^ platelets/ml) were preincubated with vehicle (NaCl 0.9%), RGDS, ^p^YMESRADR or with 317-substituted octapeptide (^p^YMESAADR), 319-substituted octapeptide (^p^YMESRAAR) or 320-substituted octapeptide (^p^YMESRADA) (500 μM) for 5 min. Platelets were then treated with 0.1 U/ml thrombin and fibrinogen-binding was measured by single-colour flow cytometry using a FITC-labeled rabbit anti-fibrinogen antibody. Results are expressed as mean fluorescence intensity of at least 3 experiments +/- SEM.
(PDF)
