# Supplementary figures and images for: Inhibition of αIIbβ3 Ligand Binding by an αIIb Peptide that Clasps the Hybrid Domain to the βI Domain of β3
Source: PLoS One. 2015 Sep 2;10(9):e0134952. doi: 10.1371/journal.pone.0134952 (PMC4557944; doi:10.1371/journal.pone.0134952)

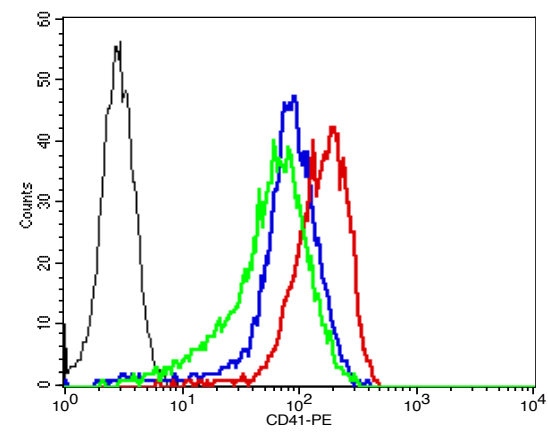

Supplement: S1 Fig — CHO cells expressing the αIIbβ3 wild-type receptor (blue line), the αIIb3Mβ3 mutant (αIIbR317A/D319A/R320A-β3) (green line) or the αIIbβ31M (αIIb-β3K384A) (red line) were labeled with saturating amounts of anti-αIIb-PE antibody. The isotype control is shown as the thin black line. (PDF) [file pone.0134952.s001.pdf]

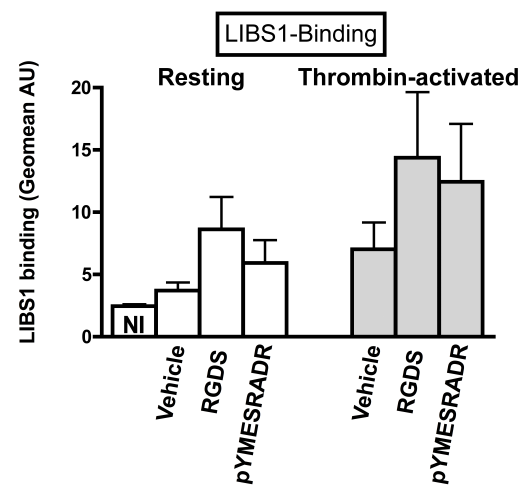

Supplement: S2 Fig — LIBS1 or control antibody (NI) binding on resting or thrombin-activated platelets pre-incubated with vehicle, RGDS or pYMESRADR peptides. Bound LIBS1 was revealed with PE- anti-mAb antibody and quantified on Becton Dickinson FACSort cytometer. (PDF) [file pone.0134952.s002.pdf]

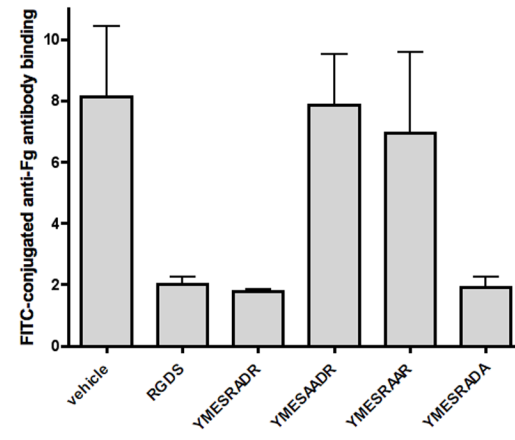

Supplement: S3 Fig — Washed platelets (2.5x10^8^ platelets/ml) were preincubated with vehicle (NaCl 0.9%), RGDS, pYMESRADR or with 317-substituted octapeptide (pYMESAADR), 319-substituted octapeptide (pYMESRAAR) or 320-substituted octapeptide (pYMESRADA) (500 μM) for 5 min. Platelets were then treated with 0.1 U/ml thrombin and fibrinogen-binding was measured by single-colour flow cytometry using a FITC-labeled rabbit anti-fibrinogen antibody. Results are expressed as mean fluorescence intensity of at least 3 experiments +/- SEM. (PDF) [file pone.0134952.s003.pdf]
